# Improving prediction accuracy of tumor classification by reusing genes discarded during gene selection

**DOI:** 10.1186/1471-2164-9-S1-S3

**Published:** 2008-03-20

**Authors:** Jack Y Yang, Guo-Zheng Li, Hao-Hua Meng, Mary Qu Yang, Youping Deng

**Affiliations:** 1 Harvard Medical School, Harvard University, Cambridge, Massachusetts 02140-0888 USA; 2 School of Computer Engineering & Science, Shanghai University, Shanghai 200072, China; 3 Institute of Systems Biology, Shanghai University, Shanghai 200072, China; 4 National Human Genome Research Institute, National Institutes of Health, U.S. Department of Health and Human Services, Bethesda, MD 20892, USA; 5 Department of Biological Sciences, University of Southern Mississippi, Hattiesburg, MS 39406, USA

## Abstract

**Background:**

Since the high dimensionality of gene expression microarray data sets degrades the generalization performance of classifiers, feature selection, which selects relevant features and discards irrelevant and redundant features, has been widely used in the bioinformatics field. Multi-task learning is a novel technique to improve prediction accuracy of tumor classification by using information contained in such discarded redundant features, but which features should be discarded or used as input or output remains an open issue.

**Results:**

We demonstrate a framework for automatically selecting features to be input, output, and discarded by using a genetic algorithm, and propose two algorithms: GA-MTL (Genetic algorithm based multi-task learning) and e-GA-MTL (an enhanced version of GA-MTL). Experimental results demonstrate that this framework is effective at selecting features for multi-task learning, and that GA-MTL and e-GA-MTL perform better than other heuristic methods.

**Conclusions:**

Genetic algorithms are a powerful technique to select features for multi-task learning automatically; GA-MTL and e-GA-MTL are shown to to improve generalization performance of classifiers on microarray data sets.

## Background

Tumor classification is performed on microarray data collected by DNA microarray experiments from tissue and cell samples [1-3]. The wealth of such data for different stages of the cell cycle aids in the exploration of gene interactions and in the discovery of gene functions. Moreover, genome-wide expression data from tumor tissues gives insight into the variation of gene expression across tumor types, thus providing clues for tumor classification of individual samples. The output of a microarray experiment is summarized as an *p* × *n* data matrix, where *p* is the number of tissue or cell samples and *n* is the number of genes. Here *n* is always much larger than *p*, which degrades the generalization performance of most classification methods. To overcome this problem, feature selection methods are applied to reduce the dimensionality from *n* to *k* with *k* <<*n*.

Feature selection chooses a subset of the original features (genes) according to the classification performance; the optimal subset should contain relevant but non-redundant features. Feature selection can help to improve the generalization performance of classifiers, and to reduce learning time and the time required to classify out-of-sample data. There has been a great deal of work in machine learning and related areas to address this issue [4-7]. In most practical cases, relevant features are selected and kept as input, while irrelevant and redundant features are removed.

Although the removed features are redundant and weakly relevant, they contain useful information that can be used to improve prediction accuracy. Multi-Task Learning (MTL) is a method of using the redundant information by selecting features from the discarded feature set to add to the target [8,9]. Although MTL achieves only limited improvement, it is nevertheless useful for real world cases like medical problems [10] and multivariate calibration problems [11].

Previous studies of search methods for multi-task learning mainly used heuristic methods [9,11], where the number of features selected for the input and/or target is somewhat arbitrary. When the search method is regarded as a combinational optimization problem, random search methods can be used. The genetic algorithm [12] is a simple and powerful method which has obtained satisfactory results for feature selection [13]. Motivated by this, we proposed the random method GA-MTL (Genetic Algorithm based Multi-Task Learning) [14], but GA-MTL did not consider irrelevant features in the data sets. Here we propose an enhanced version of GA-MTL (e-GA-MTL) which codes one feature with two binary bits. The e-GA-MTL algorithm and others are applied to tumor classification on microarray data sets; it is found that e-GA-MTL outperforms all other algorithms considered.

## Results and discussion

In order to demonstrate the benefits of multi-task learning methods, we have performed the following series of experiments using artificial neural networks (ANNs) as classifiers.

1. ALL is a baseline method; without any selection, all the genes are input to the ANN for classification.

2. GA-FS uses a genetic algorithm to select genes and input selected genes to the ANN.

3. H-MTL uses a heuristic embedded feature selection method to search features, where some of the selected features serve as input to the ANN and some of the features are added to the output.

4. GA-MTL uses a genetic algorithm to search features, where some of the selected features are input into ANN and some of the features are added to the output.

5. GA-MTL-IR uses an embedded algorithm to remove irrelevant features and then uses a genetic algorithm to search features, where some of the selected features serve as input to the ANN and some of the features are added to the output.

6. e-GA-MTL also uses a genetic algorithm to search features, and employs two bits to represent one feature; some features are considered as irrelevant and discarded, some of the selected features serve as input to the ANN, and some of the features are added to the output.

The most important parameter of an ANN is the number of nodes in hidden layer, *M*. To reduce the effect of this parameter we ran the experiments with both *M* = 2 and *M* = 10.

While different data sets, including data sets with only the selected features, need different optimal parameters for different methods, we do not try to find the optimal parameters, because:

(1) It is infeasible to find the optimal parameters, because this is an NP-hard problem.

(2) We are not interested in obtaining the best performance of one special method on a given data set; instead, we are interested in demonstrating the effect of our proposed framework.

### Prediction performance

**Figure 1:**
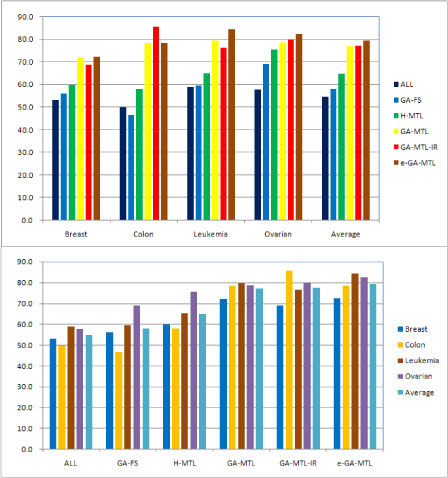
**Performance of multi-task learning algorithms for ANNs with *M* = 2 hidden units.** Both graphs show balanced accuracy (BACC) scores. Top: Results grouped by data set. Bottom: Results grouped by multi-task learning algorithm

**Figure 2:**
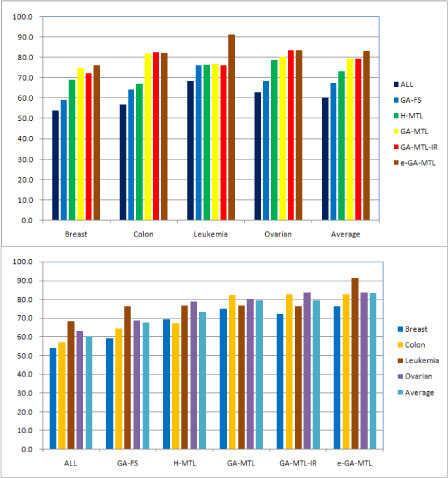
**Performance of multi-task learning algorithms for ANNs with *M* = 10 hidden units.** Both graphs show balanced accuracy (BACC) scores. Top: Results grouped by data set. Bottom: Results grouped by multi-task learning algorithm.

The average BACC values are shown in Figures 1 and 2 for different values of the ANN parameters, where ALL means all the genes are used as input for classification without any gene selection. From Figures 1 and 2, we conclude that:

(1) On average and for all the data sets, the multi-task learning algorithms H-MTL, GA-MTL, GA-MTL-IR, and e-GA-MTL perform better than the feature selection algorithms GA-FS and ALL.

(2) On average and for almost all the data sets, the genetic algorithm based multi-task learning algorithms GA-MTL, GA-MTL-IR and e-GA-MTL perform better than H-MTL, a heuristic algorithm. Only on the leukemia data set, for an ANN with *M* = 10 hidden units, does H-MTL perform slightly better than GA-MTL and GA-MTL-IR.

(3) On average, e-GA-MTL performs the best among all the learning algorithms.

(4) Although GA-FS performs worse than the multi-task learning algorithms, it performs better than those without any gene selection.

Detailed statistical values of BACC, correction, sensitivity, specificity, and precision are also listed in Tables 1,2,3,4 and 5, from which we conclude that:

**Table 1:** Mean and standard deviation (in parentheses) of BACC scores (%), calculated over 50 hold out runs.

DATASET	ALL	GA-FS	H-MTL	GA-MTL	GA-MTL-IR	e-GA-MTL
*M* = 2 for ANN						

Breast	53.2(9.3)	56.1(8.6)	59.8(8.5)	72.0(8.3)	69.0(8.4)	72.4(8.4)
Colon	50.0(8.8)	46.7(8.5)	58.1(7.9)	78.6(8.4)	85.7(8.1)	78.6(7.8)
Leukemia	59.1(7.8)	59.7(8.2)	65.2(7.9)	79.6(7.9)	76.4(7.7)	84.5(7.6)
Ovarian	57.8(6.8)	69.1(6.8)	75.6(3.8)	78.9(7.2)	79.9(7.1)	82.4(7.1)
Average	54.8(8.2)	58.1(8.0)	64.8(7.8)	77.1(7.9)	77.4(7.8)	79.5(7.7)

*M* = 10 for ANN						

Breast	54.2(9.3)	59.3(8.9)	69.2(9.2)	74.9(8.8)	72.3(9.3)	76.2(8.7)
Colon	57.0(8.9)	64.4(8.6)	67.4(9.0)	82.2(8.6)	82.7(8.6)	82.5(8.8)
Leukemia	68.4(8.1)	76.2(7.7)	76.6(8.0)	76.8(7.5)	76.4(7.1)	91.4(7.2)
Ovarian	63.0(6.3)	68.5(6.6)	78.8(5.9)	80.3(6.0)	83.5(6.2)	83.6(6.3)
Average	60.4(8.2)	67.6(8.0)	73.2(8.0)	79.4(8.0)	79.5(7.8)	83.3(7.7)

**Table 2:** Mean and standard deviation (in parentheses) of correction scores (%), calculated over 50 hold out runs.

DATASET	ALL	GA-FS	H-MTL	GA-MTL	GA-MTL-IR	e-GA-MTL
*M* = 2 for ANN						

Breast	53.1(9.5)	56.3(8.4)	59.3(8.8)	71.9(8.9)	68.8(8.1)	71.9(8.6)
Colon	57.1(7.8)	61.9(7.3)	66.6(7.1)	80.9(6.3)	85.7(6.4)	80.9(6.4)
Leukemia	57.7(9.3)	53.8(9.0)	61.5(9.5)	76.9(8.4)	76.9(9.1)	80.7(8.9)
Ovarian	57.1(6.4)	67.9(7.4)	75.0(5.4)	78.6(5.9)	79.8(5.1)	82.1(5.8)
Average	56.3(8.3)	60.0(8.0)	65.6(7.7)	77.1(7.4)	77.8(7.2)	78.9(7.4)

*M* = 10 for ANN						

Breast	64.3(8.5)	67.3(8.7)	65.7(8.3)	75.4(8.3)	71.8(7.5)	75.8(8.0)
Colon	62.0(7.5)	71.2(7.2)	75.2(6.5)	85.1(6.7)	82.9(6.1)	83.9(6.3)
Leukemia	65.3(8.8)	69.2(8.4)	76.3(8.8)	84.6(8.9)	83.1(8.1)	85.6(8.3)
Ovarian	61.9(7.5)	65.4(7.7)	78.6(6.4)	80.9(6.8)	82.1(6.4)	83.3(6.8)
Average	63.4(8.1)	68.3(8.0)	74.0(7.5)	81.5(7.7)	80.0(7.0)	82.2(7.4)

**Table 3:** Mean and standard deviation (in parentheses) of sensitivity scores (%), calculated over 50 hold out runs.

DATASET	ALL	GA-FS	H-MTL	GA-MTL	GA-MTL-IR	e-GA-MTL
*M* = 2 for ANN						

Breast	53.3(8.7)	53.3(8.5)	66.7(8.0)	73.3(7.8)	73.3(7.5)	80.0(7.6)
Colon	28.6(7.4)	28.6(7.5)	42.8(7.4)	71.4(7.0)	85.7(7.1)	71.4(7.1)
Leukemia	62.5(8.1)	75.0(8.0)	75.0(7.7)	87.5(7.9)	75.0(7.8)	85.7(7.7)
Ovarian	60.0(7.5)	73.3(7.4)	76.7(7.4)	80.0(6.7)	80.0(6.9)	83.3(7.2)
Average	51.1(7.9)	57.6(7.9)	65.3(7.6)	78.1(7.4)	78.5(7.3)	80.1(7.4)

*M* = 10 for ANN						

Breast	60.2(9.8)	63.2(9.4)	73.3(10.4)	80.5(9.5)	80.6(9.0)	85.6(9.0)
Colon	42.7(7.8)	43.4(7.5)	51.2(7.4)	72.1(7.5)	72.3(7.1)	72.3(6.8)
Leukemia	71.3(7.6)	75.0(7.5)	77.5(7.9)	88.7(8.1)	74.3(7.9)	91.2(8.3)
Ovarian	64.7(7.5)	71.4(7.3)	80.5(7.4)	81.2(7.0)	80.0(6.9)	86.7(7.1)
Average	59.7(8.2)	63.3(7.9)	70.6(8.3)	80.6(8.0)	76.8(7.7)	84.0(7.8)

**Table 4:** Mean and standard deviation (in parentheses) of specificity scores (%), calculated over 50 hold out runs.

DATASET	ALL	GA-FS	H-MTL	GA-MTL	GA-MTL-IR	e-GA-MTL
*M* = 2 for ANN						

Breast	53.0(9.9)	58.8(8.6)	52.9(9.0)	70.7(8.7)	64.7(9.2)	64.8(9.2)
Colon	71.3(10.2)	64.7(9.5)	73.4(8.4)	85.7(9.8)	85.7(9.1)	85.7(8.4)
Leukemia	55.6(7.5)	44.4(8.3)	55.4(8.1)	71.7(7.8)	77.8(7.5)	83.3(7.4)
Ovarian	55.6(6.1)	64.9(6.2)	74.4(6.1)	77.7(7.6)	79.7(7.3)	81.5(7.0)
Average	58.5(8.4)	58.6(8.1)	64.3(8.0)	76.1(8.4)	76.3(8.2)	78.8(8.0)

*M* = 10 for ANN						

Breast	48.1(8.8)	55.4(8.4)	65.1(8.0)	69.2(8.1)	63.9(9.6)	66.8(8.4)
Colon	71.3(10.0)	85.3(9.7)	83.5(10.5)	92.2(9.7)	93.1(10.0)	92.6(10.8)
Leukemia	65.4(8.5)	77.4(7.8)	75.6(8.1)	64.8(6.8)	78.4(6.2)	91.5(6.0)
Ovarian	61.3(5.1)	65.6(5.9)	77.0(4.3)	79.4(5.0)	87.0(5.4)	80.4(5.5)
Average	61.0(8.1)	71.8(8.1)	75.8(7.6)	78.1(7.9)	82.2(7.8)	82.6(7.6)

**Table 5:** Mean and standard deviation (in parentheses) of precision scores (%), calculated over 50 hold out runs.

DATASET	ALL	GA-FS	H-MTL	GA-MTL	GA-MTL-IR	e-GA-MTL
*M* = 2 for ANN						

Breast	50.0(8.8)	53.3(8.4)	55.5(8.4)	68.8(8.1)	64.7(7.9)	66.7(8.2)
Colon	33.3(8.7)	25.0(8.1)	42.9(7.4)	71.4(7.8)	75.0(7.7)	71.4(7.4)
Leukemia	38.5(7.7)	37.5(7.9)	42.8(7.5)	58.3(7.6)	60.0(7.4)	66.7(7.1)
Ovarian	42.9(6.4)	53.7(6.6)	62.6(6.1)	66.6(6.8)	68.6(7.1)	71.4(6.5)
Average	41.2(7.9)	42.4(7.8)	51.0(7.4)	66.3(7.6)	67.1(7.5)	69.1(7.3)

*M* = 10 for ANN						

Breast	42.6(9.2)	60.0(9.0)	65.4(9.7)	82.4(8.4)	82.1(9.2)	82.6(9.1)
Colon	51.2(7.5)	56.2(7.4)	64.7(7.3)	70.6(8.1)	66.7(8.2)	68.4(7.3)
Leukemia	46.1(7.8)	63.1(7.4)	63.6(7.6)	57.0(7.4)	60.0(6.4)	67.1(6.8)
Ovarian	47.6(5.4)	53.6(5.7)	66.4(4.3)	68.4(5.1)	78.2(5.0)	72.1(5.5)
Average	46.9(7.5)	58.2(7.4)	65.0(7.2)	69.6(7.3)	71.8(7.2)	72.6(7.2)

(1) Although the results for ANNs with *M* = 10 are better than those for *M* = 2, we can draw similar conclusions for both series of results in terms of how the different methods compare.

(2) For all the measures, on average, multi-task learning algorithms including H-MTL, GA-MTL, GA-MTL-IR, and e-GA-MTL perform better than GA-FS and ALL, and genetic algorithm based multi-task learning algorithms like GA-MTL, GA-MTL-IR, and e-GA-MTL perform better than H-MTL.

(3) Both e-GA-MTL and GA-MTL-IR remove irrelevant genes; both obtain better results than the others for the specificity, precision and BACC measures, on average. But GA-MTL-IR performs worse than GA-MTL for other measures like sensitivity and correction.

(4) e-GA-MTL performs the best among all the learning algorithms on average, for all the measures. It greatly improves results for the BACC, sensitivity and specificity measures.

### The number of selected features

We show the number of features selected by each algorithm in Tables 6 and 7, which also lists the number of discarded features, input features, and target features. For GA-FS, the features are selected as input or are discarded. For GA-MTL, the features are selected as input or are added to the target; no features are discarded. For H-MTL, GA-MTL-IR, and e-GA-MTL, the features are selected as input, are added to the target, or are discarded.

**Table 6:** Mean and standard deviation (in parentheses) of the number of features, calculated over 50 hold out runs, where the base learners are ANNs with M = 2 units in the hidden layer.

		Breast	Colon	Leukemia	Ovarian	Average
GA-FS	input	15564.4(2.1)	897.3(4.3)	3245.3(2.5)	10037.4(3.4)	7436.1(3.1)
	discarded	8916.6(1.5)	1103.6(3.2)	3883.6(2.7)	5116.6(3.8)	4755.1(2.8)
H-MTL	input	12547.4(6.5)	1014.0(5.4)	4007.8(2.5)	8924.7(2.5)	6623.5(4.2)
	output	4182.5(6.9)	338.0(3.2)	1335.9(3.4)	2974.9(3.4)	2207.8(4.2)
	discarded	7751.1(5.3)	648.2(4.3)	1785.3(2.6)	3254.6(2.5)	3359.8(3.7)
GA-MTL	input	15624.5(2.7)	993.3(3.3)	3324.7(2.0)	10154.2(4.4)	7524.2(3.1)
	output	8856.5(2.9)	1007.6(3.5)	3804.3(3.7)	4999.8(2.8)	4667.1(3.2)
GA-MTL-IR	input	12656.3(3.6)	877.4(4.5)	4231.6(2.9)	7895.4(3.5)	6415.2(3.6)
	output	4073.6(4.3)	474.4(4.6)	1112.1(2.7)	4004.0(3.1)	2416.0(3.7)
	discarded	7751.1(5.3)	648.2(4.3)	1785.3(2.6)	3254.6(2.5)	3359.8(3.7)
e-GA-MTL	input	12743.3(4.1)	884.7(5.2)	4296.4(2.9)	10235.2(3.6)	7954.4(4.0)
	output	4097.4(4.3)	486.4(4.4)	1175.6(2.1)	2354.4(4.5)	2028.5(3.8)
	discarded	7765.2(5.4)	660.0(5.1)	1796.2(2.1)	2449.2(3.6)	3167.7(4.0)

**Table 7:** Mean and standard deviation (in parentheses) of the number of features, calculated over 50 hold out runs, where the base learners are ANNs with M = 10 units in the hidden layer.

		Breast	Colon	Leukemia	Ovarian	Average
GA-FS	input	15042.4(3.5)	917.3(4.5)	3456.3(2.5)	9837.4(4.3)	7313.4(3.7)
	discarded	9438.6(1.5)	1082.6(2.8)	3672.6(2.9)	5316.6(3.7)	4877.6(2.7)
H-MTL	input	12620.2(5.6)	1042.3(4.5)	4082.1(3.6)	8847.3(2.1)	6648.0(4.0)
	output	4206.7(5.9)	347.5(2.3)	1360.7(3.1)	2949.1(4.3)	2216.0(3.9)
	discarded	7654.1(3.5)	610.2(2.7)	1686.3(5.2)	3357.6(2.1)	3327.1(3.4)
GA-MTL	input	15153.3(2.5)	1041.1(4.1)	3435.4(4.3)	10034.5(3.3)	7416.1(3.6)
	output	9327.7(2.7)	959.0(2.5)	3693.6(3.8)	5119.5(3.4)	4775.0(3.1)
GA-MTL-IR	input	12541.5(3.6)	842.2(4.5)	4325.6(2.8)	7984.2(2.1)	6423.4(3.3)
	output	4285.4(4.3)	547.6(4.6)	1117.1(2.0)	3812.2(3.8)	2440.6(3.7)
	discarded	7654.1(3.5)	610.2(2.7)	1686.3(5.2)	3357.6(2.1)	3327.1(3.4)
e-GA-MTL	input	12700.3(4.1)	854.7(4.1)	4147.1(2.9)	10453.2(3.5)	7038.8(3.7)
	output	4154.4(4.5)	486.4(4.4)	1272.7(2.7)	2454.4(3.5)	2092.0(3.8)
	discarded	7645.2(5.3)	660.0(5.7)	1846.2(2.4)	2489.2(3.7)	3160.2(4.3)

From Tables 6 and 7, we can see that:

(1) For GA-FS, about one third of genes are removed and two thirds are used for classification. Furthermore, the ratio of the number of input features to the number of output features for GA-MTL is similar to the ratio of the number of input features to the number of discarded features for GA-FS.

(2) H-MTL and GA-MTL-IR both use the same prediction risk criterion to discard irrelevant features, so the features discarded are the same and hence the number of discarded features are the same. The number of input features and output features are different, however. H-MTL has a predetermined number of input and output features; one quarter of the selected features are used for the input, and the other three quarters are added to the output. In contrast, for GA-MTL-IR, the features are determined by a genetic algorithm, but the ratio of the number of input features to the number of output features is similar to that of H-MTL.

(3) For e-GA-MTL, although the number of input, output and discarded features are determined automatically by the genetic algorithm, the ratios among these numbers are similar to those for H-MTL and GA-MTL-IR.

### Discussions

We have demonstrated that genetic algorithm based multi-task learning (GA-MTL) methods perform better than the heuristic methods and feature selection methods, and that e-GA-MTL performs the best of all the methods considered. Several questions come immediately to mind:

#### Why does multi-task learning succeed?

In a previous study, Caruana *et al.* gave an explanation [8,9] of why multi-task learning succeeds. Here we combine their results with the framework presented here. Yu and Liu [15] proposed to categorize the features into four classes, namely:

I: irrelevant features,

II: weakly relevant and redundant features,

III: weakly relevant but non-redundant features, and IV: strongly relevant features;

where III and IV comprise the optimal feature subset and I and II should be removed using feature selection methods. We have found that II contains useful information. These features should not be discarded, but rather should be used in the learning process. Multi-task learning is a method to use these redundant features to improve the prediction accuracy of the base learning method, which accounts for its improved performance.

#### Why do genetic algorithms perform better than the heuristic method?

Our results demonstrate that genetic algorithm based multi-task learning methods outperform heuristic multi-task learning methods. The chief reason why this is so is that the heuristic method considered here uses the feature ranking technique to select features for the input and the target, which does not consider feature redundancy and/or feature interaction. At the same time, is somewhat arbitrary to use a prespecified number of features for the input and the target. This is another factor which reduces the performance of the heuristic method. In contrast, when the genetic algorithm selects features for the input and the target, it simultaneously considers feature redundancy and/or feature interaction. So it automatically determines the number of features for the input and target. In fact, Kudo and Sklansky proved that genetic algorithms have a higher probability of finding better solutions to naive feature selection problems than other complete, heuristic and random algorithms [16]. Among the genetic algorithm based multi-task learning methods, e-GA-MTL performs better than GA-MTL-IR. The number of features removed by e-GA-MTL is determined automatically by the genetic algorithm, while the number removed by GA-MTL-IR is prespecified. This is further evidence that genetic algorithm based approaches outperform heuristic approaches.

#### What effect do irrelevant features have on multi-task learning?

The effect of multi-task learning on irrelevant features can be observed by comparing the results obtained by e-GA-MTL, GA-MTL-IR, and GA-MTL; e-GA-MTL and GA-MTL-IR remove irrelevant features, while GA-MTL does not. Here we observed that e-GA-MTL and GA-MTL-IR outperformed GA-MTL, especially for the sensitivity and BACC measures. This shows that irrelevant features will degrade the generalization performance of multi-task learning methods, and reduce the robustness of the methods; they should therefore be removed before the learning process.

## Conclusions

Random search methods of multi-task learning (MTL), including GA-MTL (Genetic Algorithm based MTL), GA-MTL-IR (GA-MTL with irrelevant features removed) and e-GA-MTL (an enhanced version of GA-MTL) are shown to improve the accuracy of multi-task learning and to make multi-task learning more convenient to use. Experimental results on microarray data sets for tumor classification showed that genetic algorithm based multi-task learning performed better than H-MTL, a heuristic multi-task learning method, and GA-FS, a naive feature selection method based on genetic algorithms. Furthermore, our results showed that e-GA-MTL and GA-MTL-IR, which remove irrelevant features, performed better than GA-MTL, which does not. e-GA-MTL, which employs a genetic algorithm with a two bit encoding to remove irrelevant features and select features for the input and output, performed best. Since analysis of microarray data sets is a high dimensional problem, our results demonstrate that multi-task learning techniques can be employed to improve prediction performance of tumor classification by using redundant genes. Furthermore, our results demonstrate that genetic algorithms can be employed to improve multi-task learning by discarding irrelevant features and by selecting the input and target features automatically; GA-MTL and e-GA-MTL are shown to to improve generalization performance of classifiers on microarray and other high-dimensional data sets.

## Methods

Multi-task learning (MTL) [8,9] is a form of inductive transfer. It is applicable to any learning method that can share some of what is learned between multiple tasks. The basic idea is to use the selected features as the input feature set and to combine the target values with some of the discarded features to form the target output.

### Previous studies

There exist several heuristic search methods for multi-task learning [9,11]. Caruana and de Sa [9] used a filter feature selection model of the cross-entropy criterion and/or an embedded model of kernel regression to rank the features, then employed the top *n*_1_ features as the input feature set, and added the top *n*_2_ of the remaining features to the target, where *n*_1_ and *n*_2_ are predefined numbers. Li *et al.*[11] employed clustering algorithms to select the features, which are first clustered using Kohonen neural networks; the features near the center of clusters are then selected as the input feature subset, and when the other unselected features are ranked according to the Euclidean distance to the input, the first few features with the least distance to the input are selected to add to the target to form the output.

#### H-MTL

H-MTL (Heuristic Multi-Task Learning) is a heuristic method with embedded feature selection that is based on the work of Caruana and de Sa [9]. The embedded model employs the prediction risk criteria [17,18], which evaluates features by computing the change in training accuracy when the features are replaced by their mean values:

$$S_{i}\text{=ATR}-{\text{ATR(}\bar{\text{x}}}^{\text{i}}\text{)}$$

where ATR is the training accuracy. ATR$\left({\bar{x}}^{i}\right)$ is the test accuracy on the training set defined by:

$$ATR\left({\bar{x}}^{i}\right)=\frac{1}{p}\sum_{j=1}^{p}\left(\tilde{y}\left(x_{j}^{1},\ldots ,{\bar{x}}^{i},\ldots ,x_{j}^{n}\right)==y_{j}\right),$$

where *n* is the number of features, *p* is the number of instances, ${\bar{x}}^{i}$ is the mean value of the *i*^th^ feature, and $\tilde{y}\left(\right)$ is the prediction value of the *j*^th^ example with the *i*^th^ feature replaced by its mean value. The features with zero value are removed, since these features are not useful for learning.

**Figure 3:**
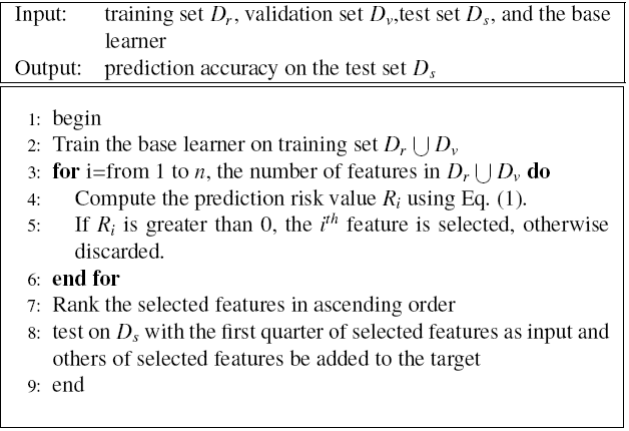
Heuristic multi-task learning (H-MTL)

After the features with zero value are removed, the prediction risk criteria is used to rank the remaining features in ascending order; the top quarter of these features are added to the output, and the remaining three quarters are used as the input. The overall algorithm is summarized in Figure 3.

#### GA-FS

To show the effectiveness of multi-task learning methods, we also implemented a naive feature selection method named GA-FS (Genetic Algorithm based Feature Selection). In GA-FS, we use a binary chromosome with the same length as the feature vector, which equals 1 if the corresponding feature is selected for the input, and 0 if the feature is discarded. The fitness function is defined as

$$fitness=\frac{1}{3}ATR+\frac{2}{3}ATV$$

where ATR is the training accuracy of the base learning method, and ATV is the prediction accuracy on the validation data set.

**Figure 4:**
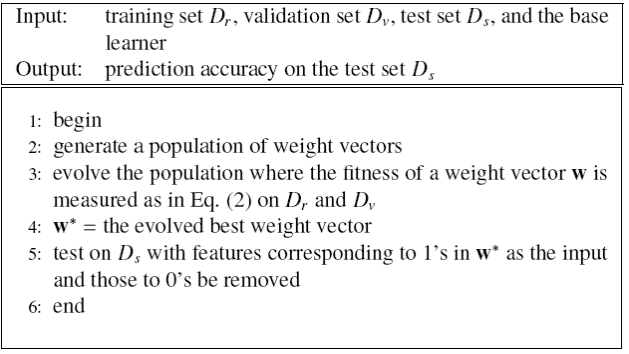
Genetic algorithm based feature selection (GA-FS)

The data set is divided into three parts: the training set *D_r_*, the validation set *D_v_*, and the test set *D_s_*, as described in the Experimental Settings subsection below. The GA-FS approach is summarized in Figure 4.

### Genetic algorithm based multi-task learning

In this subsection, we describe multi-task learning methods based on genetic algorithms. We previously proposed GA-MTL (Genetic Algorithm Based Multi-Task Learning), which did not consider irrelevant features. Here we propose two additional algorithms: GA-MTL-IR (GA-MTL with Irrelevant features Removed) and e-GA-MTL (an enhanced version of GA-MTL). GA-MTL-IR removes irrelevant features using an embedded feature selection method as in H-MTL, while e-GA-MTL removes irrelevant features using a genetic algorithm.

#### GA-MTL

In existing search methods [9,11], the number of features selected for the input and/or the target is decided somewhat arbitrarily. In order to improve feature selection, GA-MTL (Genetic Algorithm based Multi-Task Learning) [10,14], a random method, employs a genetic algorithm [12] which simultaneously selects the features for both the input and the target. The number of features for the input and target is automatically determined by the method itself. In both GA-MTL and GA-FS, the same genetic algorithm is used for the feature selection task. The only difference between GA-MTL and GA-FS is the value of the binary chromosome; in GA-MTL, it equals 0 if the feature is selected to add to the output, whereas in GA-FS, it equals 0 if the feature is removed.

**Figure 5:**
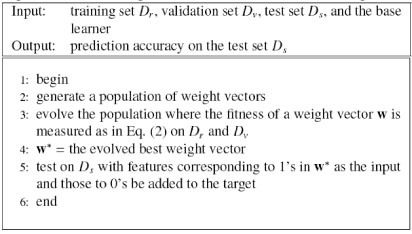
Genetic algorithm based multi-task learning (GA-MTL)

GA-MTL uses the fitness function defined by Equation (1). The data set is divided into three parts: the training set *D_r_*, the validation set *D_v_*, and the test set *D_s_*, as described in the Experimental Settings subsection below. The GA-MTL algorithm is summarized in Figure 5.

#### GA-MTL-IR

**Figure 6:**
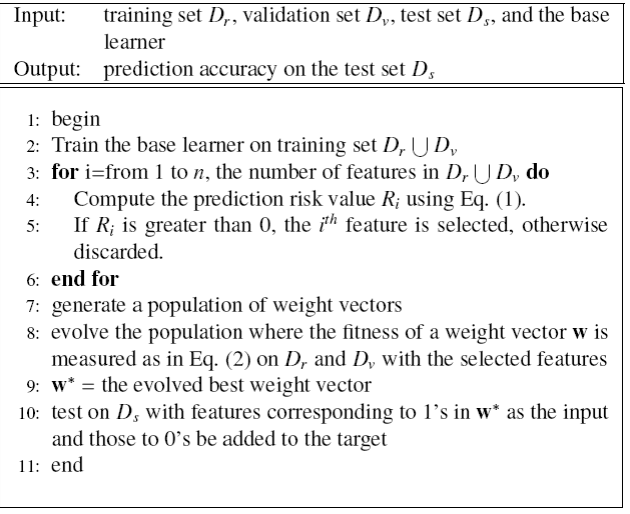
GA-MTL with irrelevant feature removed (GA-MTL-IR)

In GA-MTL, the irrelevant features are still present. These can be removed by many feature selection methods [6]. Here, we consider using the prediction risk criterion [17,18] in an embedded method. As shown in Figure 6, first the features with a prediction risk value of zero are removed, then GA-MTL is performed on the data set with the selected features. As this method removes the irrelevant features for GA-MTL, it is named GA-MTL-IR (GA-MTL with Irrelevant features Removed).

#### e-GA-MTL

**Figure 7:**
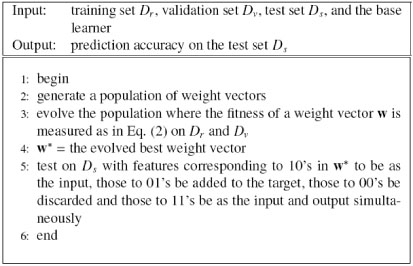
Enhanced version of GA-MTL (e-GA-MTL)

GA-MTL-IR removes irrelevant features using an embedded method, but it searches features for MTL using a genetic algorithm. Thus two search algorithms are used in GA-MTL-IR; why not instead use only a genetic algorithm? We propose an enhanced version of GA-MTL (e-GA-MTL), which is summarized in Fig. 7. It difiers from GA-MTL in its binary chromosome; instead of only one bit, two bits are used to represent each feature, where 00 means the corresponding feature is discarded, 10 means it is used as input, 01 means it is added to the output, and 11 means it is used as input and added to the output simultaneously.

#### The base learning method

Since artificial neural networks are a frequently used and powerful learning method, improved multi-layer perception neural networks were used as the base learning method. These are weight decay based neural networks in a Bayesian framework, which adds a regularization term to the objective function and are to some degree insensitive to the parameter settings [19].

### Experimental data sets

The eight microarray data sets used in our study are listed in Table 8, and are briefy described below. Versions of the data files formatted for C4.5 are available [20].

**Table 8:** Microarray data sets used for comparison

Data Sets	Samples	Class Ratio	Features

Breast Cancer	97	46/51	24,481
Colon	62	22/40	2,000
Leukemia	72	25/47	7,129
Ovarian	253	91/162	15,154

**Breast Cancer:** Van't Veer *et al.*[21] used DNA microarray analysis on primary breast tumors and applied supervised classification methods to identify significant genes for the disease. The data contains 97 patient samples, 46 of which are from patients who had developed distance metastases within 5 years (labeled as “relapse”), the remaining 51 samples are from patients who remained free from the disease after their initial diagnosis for an interval of at least 5 years (labeled as “non-relapse”). The number of genes is 24,481 and the missing values of “NaN” are replaced with 100.

**Colon:** Alon *et al.*[22] used Affymetrix oligonucleotide arrays to monitor expression levels of over 6,500 human genes from 40 tumor and 22 normal colon tissue samples. The 2,000 genes with the highest minimal intensity across the 62 tissues were used in the analysis.

**Leukemia:** The acute leukemia data set, published by Golub *et al.*[23], consists of 72 bone marrow samples with 47 ALL and 25 AML. The gene expression intensities are obtained from Affymetrix high-density oligonucleotide microarrays containing probes for 7,129 genes.

**Ovarian:** Petricoin *et al. *[24] identified proteomic patterns in serum to distinguish ovarian cancer from non-cancer. The proteomic spectral data includes 91 controls (Normal) and 162 ovarian cancers; each sample contains the relative amplitude of the intensity at 15,154 molecular mass/charge (M/Z) identities.

### Experimental settings

To evaluate the performance of the proposed approach, we use the hold out validation procedure. Each data set is used in its entirety, where split data sets are merged, and then the entire data set is randomly split into a training set and a testing set *D_s_*; 2/3 of the data is used for training and 1/3 for testing. If a validation set is required, the training set is further split so that 2/3 of the original training set is retained for training (forming the set *D_r_*) and 1/3 of the original training set is used for validation (forming the set *D_v_*). classification results are reported for the test data sets *D_s_*. This process is repeated 50 times.

The parameters of the genetic algorithms were set by default as in the MATLAB software, and we varied the parameters of the artificial neural networks to see how the settings of these parameters affected the results.

### Measures

In order to precisely characterize the performance of different learning methods, we define several performance measures below (see [25]). Here TP, TN, FP, and FN, stand for the number of true positive, true negative, false positive, and false negative samples, respectively.

Sensitivity is defined as $\frac{TP}{TP+FN}$ and is also known as Recall.

Specificity is defined as $\frac{TN}{TN+FP}$.

BACC (Balanced Accuracy) is defined as $\frac{1}{2}\left(\frac{TP}{TP+FN}+\frac{TN}{TN+FP}\right)$, which defines the average of sensitivity and specificity.

Precision is defined as $\frac{TP}{TP+FP}.$

Correction is defined as $\frac{TP+TN}{TP+TN+FP+FN}$ and measures the overall percentage of samples correctly classified.

## Competing interests

The authors declare that they have no competing interests.

## Authors' contributions

Jack Y. Yang conceived and guided the project; Guo-Zheng Li proposed the idea; designed the algorithm and wrote the paper; Hao-Hua Meng performed the experiments; Youping Deng helped Guo-Zheng Li in writing the paper; Mary Qu Yang provided advice and help in designing the experiments.
